# Deep learning-based single-shot phase retrieval algorithm for surface plasmon resonance microscope based refractive index sensing application

**DOI:** 10.1038/s41598-021-95593-4

**Published:** 2021-08-11

**Authors:** Kitsada Thadson, Sarinporn Visitsattapongse, Suejit Pechprasarn

**Affiliations:** 1 Department of Biomedical Engineering, School of Engineering, King Mongkut’s Institute of Technology Ladkrabang, Bangkok, 10520 Thailand; 2 Shenzhen Key Laboratory for Micro-Scale Optical Information Technology, Nanophotonics Research Center, Institute of Microscale Optoelectronics, Shenzhen University, Shenzhen, 518060 China; 3 College of Biomedical Engineering, Rangsit University, Pathum Thani, 12000 Thailand

**Keywords:** Optical sensors, Imaging and sensing, Microscopy, Interference microscopy, Nanophotonics and plasmonics

## Abstract

A deep learning algorithm for single-shot phase retrieval under a conventional microscope is proposed and investigated. The algorithm has been developed using the context aggregation network architecture; it requires a single input grayscale image to predict an output phase profile through deep learning-based pattern recognition. Surface plasmon resonance imaging has been employed as an example to demonstrate the capability of the deep learning-based method. The phase profiles of the surface plasmon resonance phenomena have been very well established and cover ranges of phase transitions from 0 to 2π rad. We demonstrate that deep learning can be developed and trained using simulated data. Experimental validation and a theoretical framework to characterize and quantify the performance of the deep learning-based phase retrieval method are reported. The proposed deep learning-based phase retrieval performance was verified through the shot noise model and Monte Carlo simulations. Refractive index sensing performance comparing the proposed deep learning algorithm and conventional surface plasmon resonance measurements are also discussed. Although the proposed phase retrieval-based algorithm cannot achieve a typical detection limit of 10^–7^ to 10^–8^ RIU for phase measurement in surface plasmon interferometer, the proposed artificial-intelligence-based approach can provide at least three times lower detection limit of 4.67 × 10^–6^ RIU compared to conventional intensity measurement methods of 1.73 × 10^–5^ RIU for the optical energy of 2500 pJ with no need for sophisticated optical interferometer instrumentation.

## Introduction

**Figure 1:**
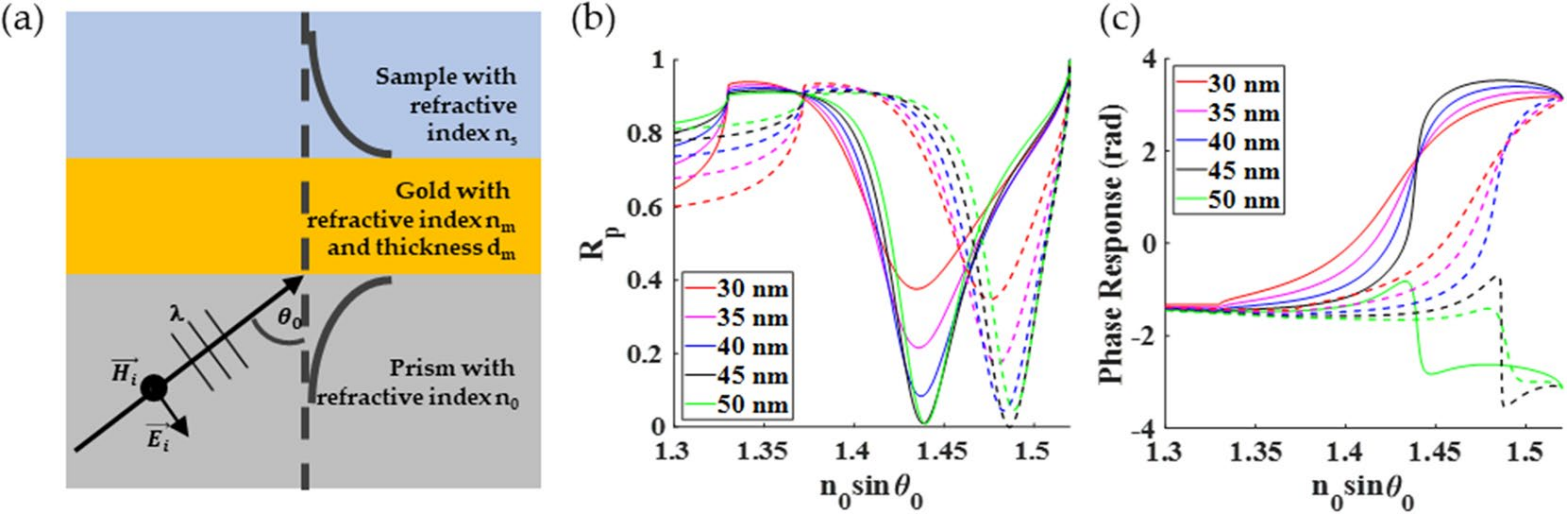
(**a**) Schematic diagram of Kretschmann configuration, (**b**) simulated reflectance for SPR gold sensors with thicknesses d_m_ of 30 nm, 35 nm, 40 nm, 45 nm, and 50 nm, and (**c**) phase responses in rad of the SPR sensors, when they were illuminated by p-polarized light at 633 nm. Solid curves for sample refractive index of 1.33 (water) and dashed curves for sample refractive index of 1.37 (liquid bovine serum albumin protein)^11^.

Surface plasmon resonance (SPR) is a resonant oscillation of electrons on the surface of noble metals when the metal surface is illuminated by p-polarized light momentum matched to the resonant condition. For biosensing applications, the Kretschmann configuration^1^, as shown in Fig. 1a, is usually employed. The system consists of a glass prism, a coherent light source in red light or infrared wavelength, index matching oil, and a 40–50 nm gold-coated coverslip. The coupling between the incident light and the SPR appears as a dark band in the reflectance spectrum due to the coupling process’s loss mechanisms^2^. The minimum intensity position in reflectance spectra is called plasmonic dip, and the incident angle corresponding to the plasmonic dip position is called plasmonic angle, ${\theta }_{p}$. The SPR is sensitive to the surrounding medium at around 200 nm from the metal surface due to its evanescent wave^3^. The SPR has been utilized and employed in a wide range of biomedical applications, including biomolecular interactions monitoring^4,5^, immunoassays^6^, DNA hybridization^7^, voltage sensing across thin membranes^8^, and binding kinetics^9,10^.

Figure 1b,c shows simulated SPR reflectance spectra and corresponding phase responses of gold thin films with different thicknesses when the gold films were illuminated with p-polarized light at 633 nm wavelength calculated using Fresnel’s equations and transfer matrix approach. It has been established that the SPR phase-detection provides a lower detection limit of 10^–7^ to 10^–8^ refractive index unit (RIU) compared to SPR intensity detection^12^ due to 3 reasons (1) the phase has a steeper response than the amplitude or intensity, as shown in Fig. 1c. (2) The phase is more tolerant to noise than the amplitude, and (3) the phase-detection allows easier signal processing. However, an interferometer is required to make a phase measurement^13^. Although optical interferometers provide a more sensitive measurement^14^, they require a more sophisticated and precise optical alignment^15^ and a well-controlled environment^16^, such as vibration isolation^13^, reference channel^3^, and temperature control unit^17^.

Recently, computational phase retrieval algorithms (PR) have been of interest to the optical science community. The phase retrieval algorithms^18^ have been adopted from X-ray phase retrieval^19^. The PR has opened up opportunities for new imaging modalities^20^, such as computational microscopy^21,22^ and superresolution microscopy^23^. Furthermore, the PR does not require an interferometric configuration. Therefore, this provides an opportunity to overcome challenges in optical interferometry and apply phase retrieval algorithms in optical phase imaging and optical phase measurement for sensing applications^24^.

Another recent advancement in computational imaging is deep learning (DL), utilizing artificial neural networks to improve several imaging techniques, such as classification^25^, segmentation^26^, and regression^27^. These data-driven algorithms have been used in optical phase imaging^28^ and superresolution microscopy^29^. However, there is still no quantitative measure to assess the performance and reliability of the phase or data recovered from the DL^30^.

Here, we propose a deep learning-based phase retrieval method with experimental verification for conventional optical microscope configuration and a theoretical framework to quantify output phases from the proposed method and compare its performance with measurement techniques in the literature. The SPR has been employed as an example for phase retrieval. The phase responses of the SPR are established^3,31^, and they cover a wide range of phase gradients, phase shifts from below 2π to 2π rad, phase positions, and phase transition directions due to the loss mechanism of the SPR^2^, as shown in Fig. 1c. Simulated data for different optical detection schemes are employed to (1) train the proposed DL-based phase retrieval method and (2) quantify phase profile, analyze the proposed DL-based phase retrieval method’s performance, and (3) compare the performance with well-known methods in the literature using the Monte Carlo-based shot noise model.

This paper demonstrates that optical phase imaging can be achieved through deep learning using pattern recognition of a single-shot intensity image captured under a conventional microscope configuration with no need for sophisticated interferometer instrumentation and a computational retrieval algorithm. To the best of the author’s knowledge, systematically quantifying the DL-based phase retrieval method and experimental validation of the proposed DL-based method have never been reported before in the literature.

## Materials and methods

### Surface plasmon microscope

**Figure 2:**
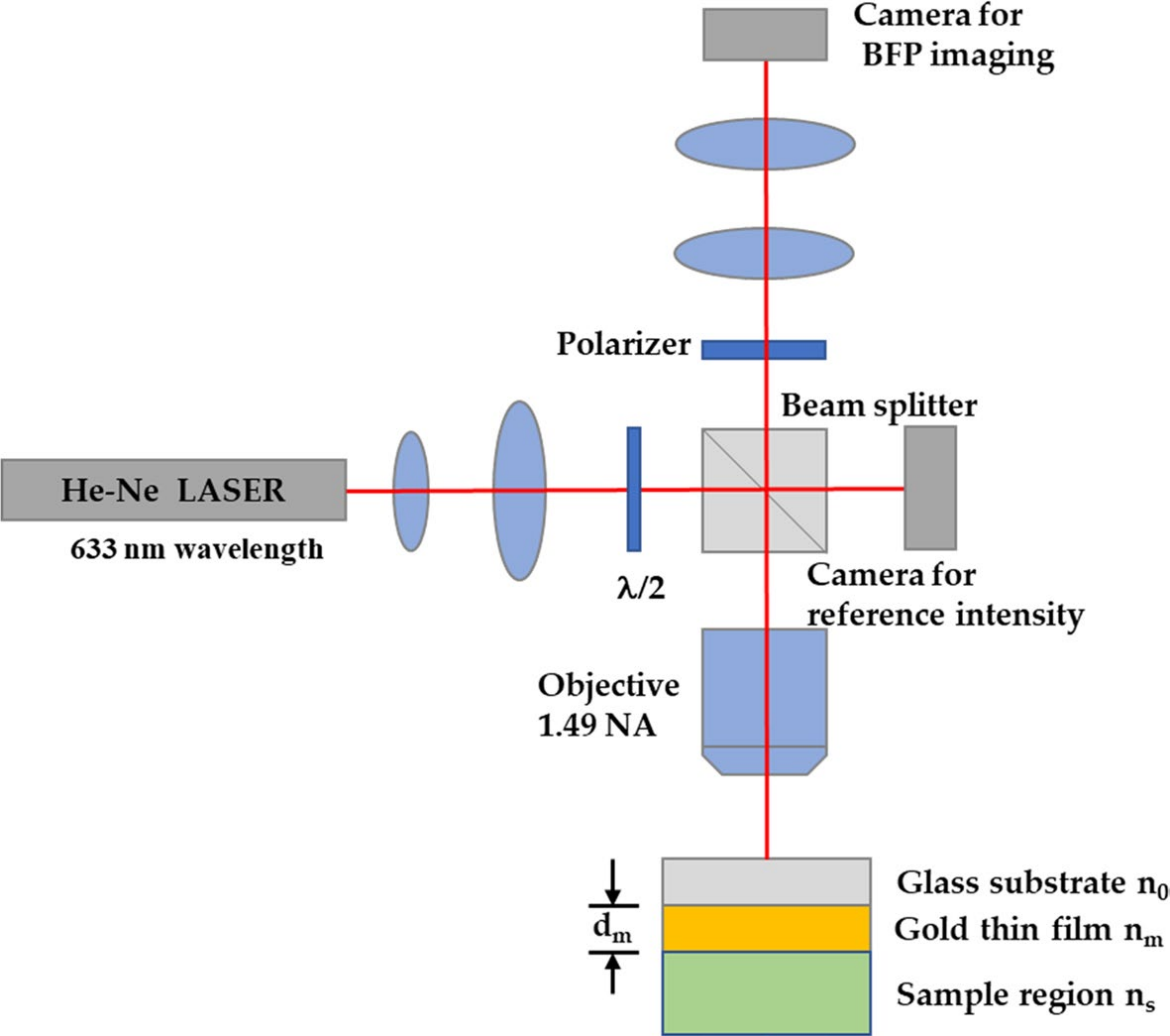
Modified optical microscope for SPR phase imaging.

The SPR phenomenon can be applied as an imaging technique called SPR microscopy (SPRM)^2,32,33^ or SPR imaging (SPRI)^34–36^, as depicted in Fig. 2. The method is used for binding^10^ or sensing events monitoring on the other side of the metallic surface with high sensitivity. This method provides real-time detection and a label-free technique.

The phase information can provide higher sensitivity and high resolution based on optical interferometry to improve the imaging technique^37,38^. The sample defocus technique^15^ is also employed to measure the phase of the SPR through an embedded optical interferometer.

The conventional microscope system can be modified to image several planes, including the image plane and the Fourier plane. Figure 2 shows a modified SPR microscope system employed in this study to quantify the performance of the proposed DL-based method. In addition, the microscope has been implemented to capture BPF images to validate the proposed network and ensure that it is also feasible to recover the phase profile in the actual experimental context, although the network was trained using the simulated data. The optical system consists of a coherent light source of the He–Ne laser at 633 nm wavelength (10 mW He–Ne laser Thorlabs), an oil immersion high numerical aperture objective (1.49NA Olympus), the gold thin film sample prepared for 46 nm of uniform gold layer coated on a BK7 glass substrate coverslip (No.0 Sigma-Aldrich) using electron beam sputterer, one 50:50 beam splitter (non-polarizing beam splitter Thorlabs), lenses (Aspherical doublet lenses Thorlabs) for beam expansion and projection, tube lens (180 mm focal length Thorlabs), the polarizer (Thorlabs) for selecting the polarization in x-axis and y-axis, and two CCD cameras (8051M-USB Thorlabs). A range of incident angles sinθ_0_ illuminates a uniform SPR gold sensor on a glass substrate provided by an oil immersion objective lens with a 1.49 numerical aperture (NA), n_0_sinθ_0_. The first camera is for capturing a reference optical intensity in the microscope. The other camera images the back focal plane (BFP) of the objective lens.

### Context aggregation network

**Figure 3:**
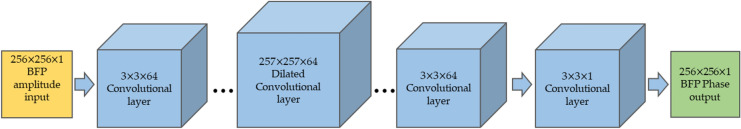
Shows the CAN network with a single BFP image input (256 pixels $\times$ 256 pixels), one BFP phase output (256 pixels $\times$ 256 pixels), and hidden layers.

Context Aggregation Network (CAN) architecture^39^ was employed for phase retrieval in this study. Generally, CAN is applied for image processing operations, such as denoising^40^, superresolution^41^, deblurring^42^, and image filtering processing^43^. The CAN network architecture shown in Fig. 3 has the input size the same as the output, and its hidden layers are highly adaptable.

The CAN network has been implemented for SPR phase retrieval with a single BFP image input (256 pixels $\times$ 256 pixels) and one BFP phase output (256 pixels $\times$ 256 pixels) shown in Fig. 3. Note that the phase in the BFP was retrieved instead of the image plane; this is to compare the SPR phase profile with conventional SPR measurement techniques reported in the literature, in which the SPR reflectance dip appears in the BFP. Of course, the proposed DL phase retrieval is not limited to only the BFP, but it is also applicable to the image plane. The completed details of the implemented CAN network are listed in Table 1 below.

**Table 1:** Specification of the CAN64.

Layer	Activations	Learnable variables	Descriptions
Image input	256 $\times$ 256 $\times$ 1	–	256 $\times$ 256 $\times$ 1 image
Convolutional	256 $\times$ 256 $\times$ 64	Weights 3 $\times$ 3 $\times$ 1 $\times$ 64, Bias 1 $\times$ 1 $\times$ 64	1 padding, 1 stride
Adaptive normalization		Offset 1 $\times$ 1 $\times$ 64, Scale 1 $\times$ 1 $\times$ 64	–
Leaky ReLU		–	Scale 0.2
Convolutional		Weights 3 $\times$ 3 $\times$ 64 $\times$ 64, Bias 1 $\times$ 1 $\times$ 64	2 padding, 1 stride, 2 dilation
Adaptive normalization		Offset 1 $\times$ 1 $\times$ 64, Scale 1 $\times$ 1 $\times$ 64	–
Leaky ReLU		–	Scale 0.2
Convolutional		Weights 3 $\times$ 3 $\times$ 64 $\times$ 64, Bias 1 $\times$ 1 $\times$ 64	4 padding, 1 stride, 4 dilation
Adaptive normalization		Offset 1 $\times$ 1 $\times$ 64, Scale 1 $\times$ 1 $\times$ 64	–
Leaky ReLU		–	Scale 0.2
Convolutional		Weights 3 $\times$ 3 $\times$ 64 $\times$ 64, Bias 1 $\times$ 1 $\times$ 64	8 padding, 1 stride, 8 dilation
Adaptive normalization		Offset 1 $\times$ 1 $\times$ 64, Scale 1 $\times$ 1 $\times$ 64	–
Leaky ReLU		–	Scale 0.2
Convolutional		Weights 3 $\times$ 3 $\times$ 64 $\times$ 64, Bias 1 $\times$ 1 $\times$ 64	16 padding, 1 stride, 16 dilation
Adaptive normalization	256 $\times$ 256 $\times$ 64	Offset 1 $\times$ 1 $\times$ 64, Scale 1 $\times$ 1 $\times$ 64	–
Leaky ReLU		–	Scale 0.2
Convolutional		Weights 3 $\times$ 3 $\times$ 64 $\times$ 64, Bias 1 $\times$ 1 $\times$ 64	32 padding, 1 stride, 32 dilation
Adaptive normalization		Offset 1 $\times$ 1 $\times$ 64, Scale 1 $\times$ 1 $\times$ 64	–
Leaky ReLU		–	Scale 0.2
Convolutional		Weights 3 $\times$ 3 $\times$ 64 $\times$ 64, Bias 1 $\times$ 1 $\times$ 64	64 padding, 1 stride, 64 dilation
Adaptive normalization		Offset 1 $\times$ 1 $\times$ 64, Scale 1 $\times$ 1 $\times$ 64	–
Leaky ReLU		–	Scale 0.2
Convolutional		Weights 3 $\times$ 3 $\times$ 64 $\times$ 64, Bias 1 $\times$ 1 $\times$ 64	128 padding, 1 stride, 128 dilation
Adaptive normalization		Offset 1 $\times$ 1 $\times$ 64, Scale 1 $\times$ 1 $\times$ 64	–
Leaky ReLU		–	Scale 0.2
Convolutional		Weights 3 $\times$ 3 $\times$ 64 $\times$ 64, Bias 1 $\times$ 1 $\times$ 64	1 padding, 1 stride
Adaptive normalization		Offset 1 $\times$ 1 $\times$ 64, Scale 1 $\times$ 1 $\times$ 64	–
Leaky ReLU		–	Scale 0.01
Convolutional	256 $\times$ 256 $\times$ 1	Weights 1 $\times$ 1 $\times$ 64, Bias 1 $\times$ 1	0 padding, 1 stride
Regression	–	–	Mean square error

The CAN uses an adaptive normalizer that can adapt its weights and biases. It can be computed with the adaptive momentum (Adam) optimizer^44^. The CAN model has ten layers; layers 1 to 8 are the dilation filters and padding functions, which their sizes increase exponentially. The output from each layer will be the same size. Layer 8 is for 128 dilation and padding functions meaning that the size of the receptive field in this layer is equal to the input image dimension. Layer 9 is for a 3 $\times$ 3 filter size, one dilation filter, and one padding function. The last convolutional layer transforms the output to the same channel size as the input using the convolutional layer with a 1 $\times$ 1 filter size and zero padding function. The last layer is the regression layer providing the predicted phase output.

The CAN model is trained under the MATLAB2019c environment with a single GPU NVIDIA GeForce GTX 1050. The network was trained with a 0.0001 learning rate and a training iteration of 50 epochs.

### Simulated dataset for training, validation, and testing

**Figure 4:**
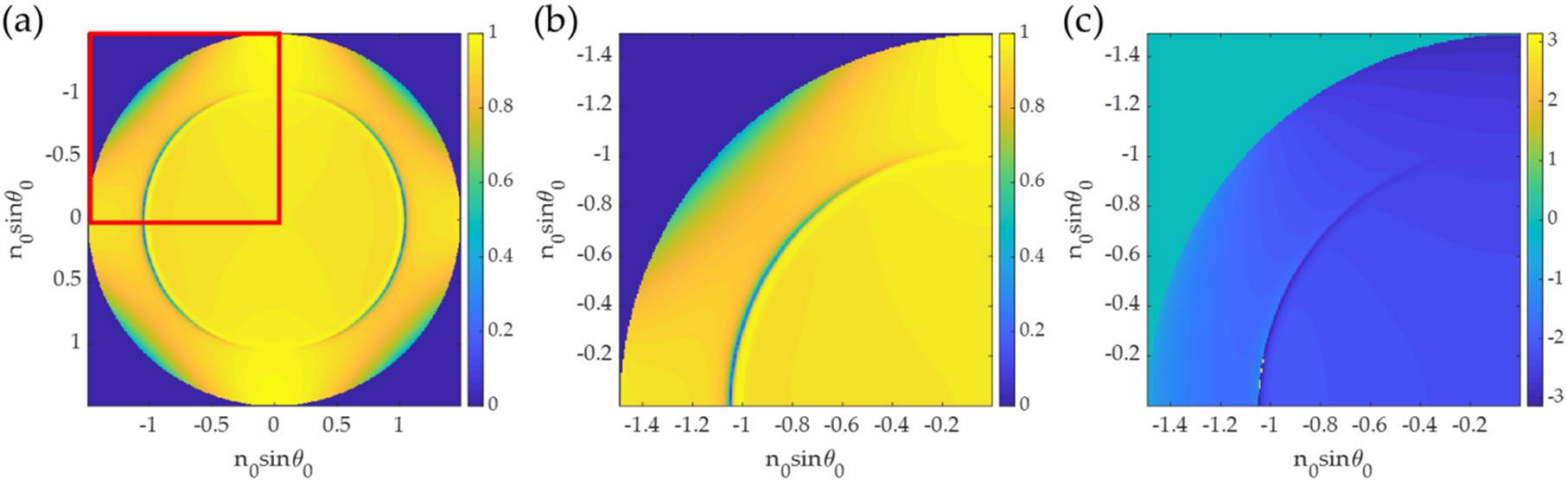
Simulated data for n_0_ of 1.52, n_m_ of 0.18 + 3.44i, d_m_ of 45 nm, n_s_ of 1.00, and l_0_ of 633 nm for (**a**) full BFP amplitude image (512 $\times$ 512 pixels) before cropping, (**b**) BFP amplitude input (256 pixels $\times$ 256 pixels), and (**b**) BFP phase output (256 pixels $\times$ 256 pixels).

Here, simulated data were employed to train the CAN network. It will be shown later that the trained network can be employed to analyze experimental data and provide the expected phase profile. The dataset simulation is based on Fresnel’s equation^31^ and the transfer matrix approach^31,45^, calculating the reflection and transmission coefficients ﻿(Fig. S1)﻿. Materials’ parameters, including the gold refractive index (n_m_) and the thickness (d_m_), the sample refractive index (n_s_), were varied to generalize the SPR phenomenon for network training accommodating for errors and discrepancies in experimental measurements. The parameters consist of random gold film thickness in a range of 20 to 60 nm (Fig. S2), random incident wavelength in a range of 550 to 650 nm, the refractive index of the gold^46^ n_m_ of 0.18 + 3.44i with ± 10% error in real part and imaginary part, and random refractive index of the sample n_s_ in a range of 1.0 to 1.4 as labeled in Fig. 2. The simulated BFP was cropped to only one quadrant (256 $\times$ 256 pixels), as shown in Fig. 4. All four quadrants carry redundant information due to symmetry in the BFP of the uniform sample. There were 1000 input and output image pairs in each dataset for training and validation. The dataset was further separated to 90% and 10% ratio for training and validation, respectively.

The dataset consists of 2 types of simulated images, including the BFP image input data and the corresponding phase output of the BFP. The phase profiles of the BFP were employed as the label for supervised learning.

**Figure 5:**
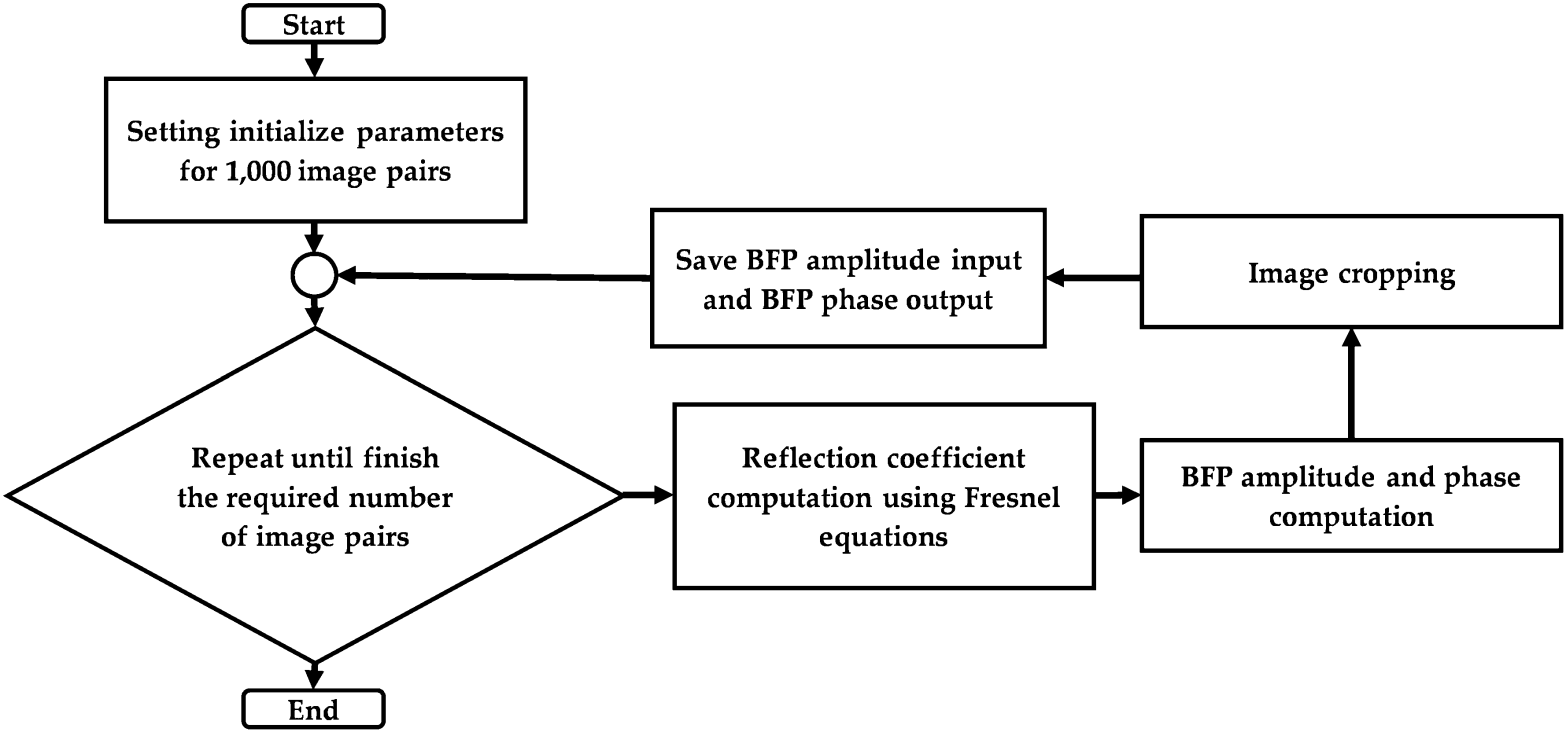
Dataset preparation flowchart.

The dataset preparation process is shown in Fig. 5. Firstly, the amplitude of BFP and phase of BFP were computed using the Fresnel equations and the transfer matrix approach. These images were then read during CAN network training and validation.

For testing the networks, the gold thicknesses of 30 nm, 40 nm, 45 nm, and 50 nm, the incident wavelength of 633 nm, and the sample refractive index range from 1.00 (air) to 1.372 (liquid BSA-protein^11^) were excluded from the dataset for training and validation to test the performance of the trained network.

### Monte Carlo based shot noise model

To estimate the phase noise of the BPF phase profile recovered from the DL-based method, the number of photons with associated shot noise in each camera pixel. The other noise sources, such as white background noise and interferences, were excluded from the consideration. From an electronic point of view, achieving the shot-noise limit is achievable, and the model can be a general performance indicator^17^. The shot noise model^17^ was employed to model the noise level using the Poisson distribution^47^. Shot noise occurs when a pixel of an imaging sensor is measured at a low light level, such as around the plasmonic dip. The shot noise is the baseline for low light intensity measurements, and it is a good indicator for the sensitivity and detection limits of measurements. The shot noise level is described by a square root of the energy detected in a digital camera pixel ($\sqrt{E}$), and the signal to noise ratio of the photon energy captured (*E*) is also the square root of the photon energy captured ($\sqrt{E}$)^17^. The test dataset explained in the “Materials and methods” section was shot noise added based on varied total photon energy in each image. The total energy of the image was varied from 90 to 2600 pJ to test the performance of the DL-based phase retrieval and the SPR dip measurement techniques. Note that camera quantum efficiency of 60% was taken into account in the shot noise computation^17^; the quantum efficiency is a typical value for a standard CCD camera^48^. Monte Carlo simulation^49^ was implemented to quantify the mean and variance of the SPR measurements for different noise levels and measurement techniques.

### SPR measurement techniques

#### Deep learning-based method

**Figure 6:**
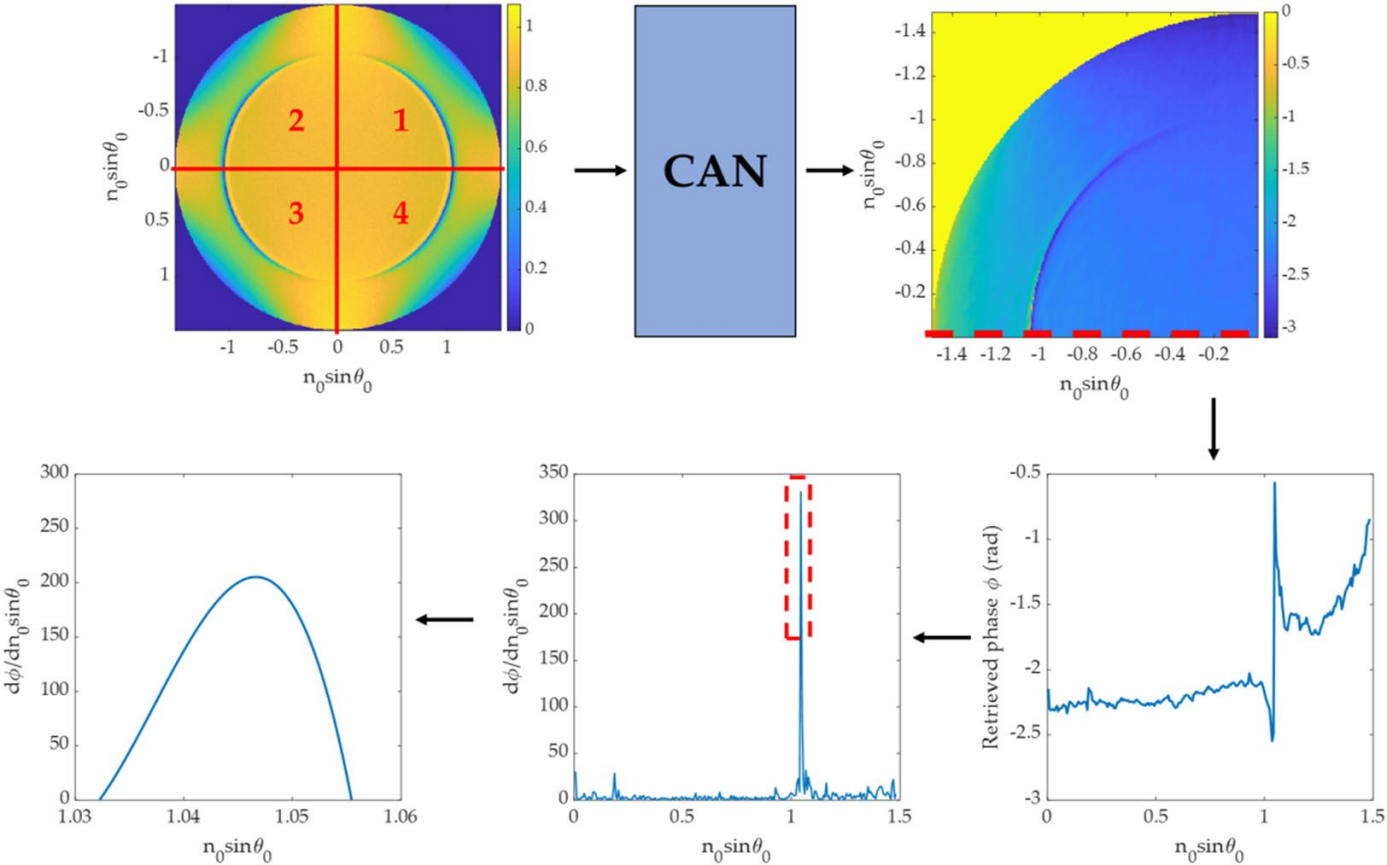
Steps in determining plasmonic angle for the DL-based method.

The trained DL network was applied to recover the one quadrant phase image, then a phase line-scan (ϕ) across the pure p-polarization as shown in Fig. 6. was employed in the later step for determining the plasmonic angle, ${\theta }_{p}$. Local gradients dϕ/dn_0_sinθ_0_ of the line scan were then computed, and the maximum local gradient position was identified. The polynomial degree 3 curve fitting was then computed around the highest dϕ/dn_0_sinθ_0_ gradients to locate the plasmonic angle from the retrieved phase profile. Note that for one BFP image, each quadrant of the BFP can be phase retrieved separately, leading to 4 output phase profiles, in which the plasmonic angles from the 4 phase outputs were then averaged and stored to compare with other measurement methods as shown in Fig. 6.

#### Polynomial degree 3 curve fitting to BFP line-scan intensity

**Figure 7:**
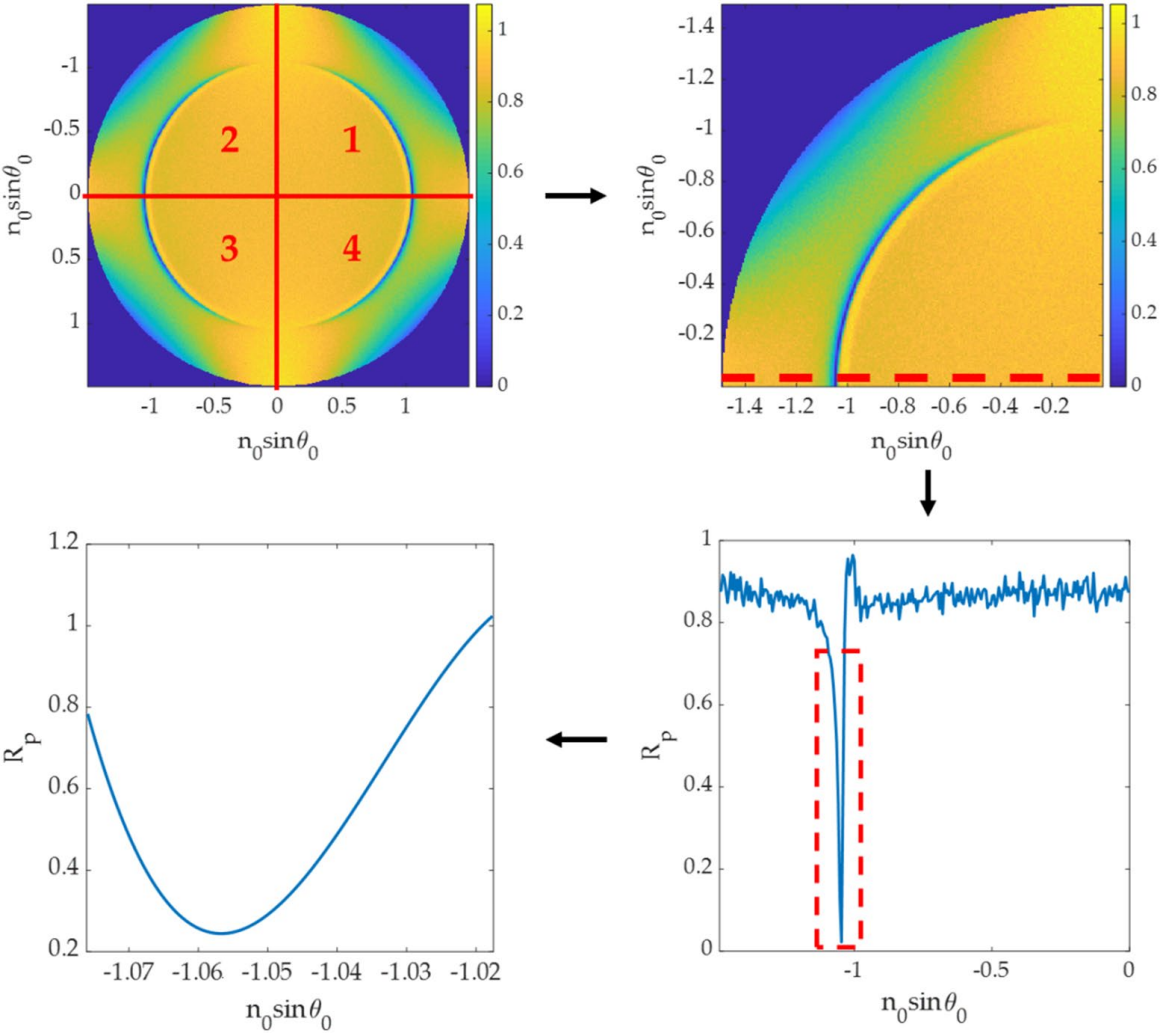
Steps in determining plasmonic angle from a BFP intensity.

This method is a conventional SPR dip measurement method from the reflectance spectrum, as shown in Fig. 7. First, a line-scan curve was extracted from the pure p-polarization axis for SPR dip position measurement. Next, the minimum intensity in the line scan was fitted using a 3rd-degree polynomial curve fitting^10^ to locate the plasmonic angle. Similar to the dip position measurement explained in the earlier section, a BFP image can then separated into four quadrants leading to 4 plasmonic angles. The mean value of the four plasmonic angles was then computed to reduce the method's measurement error and fully utilize the image.

#### Azimuthal angle averaging

**Figure 8:**
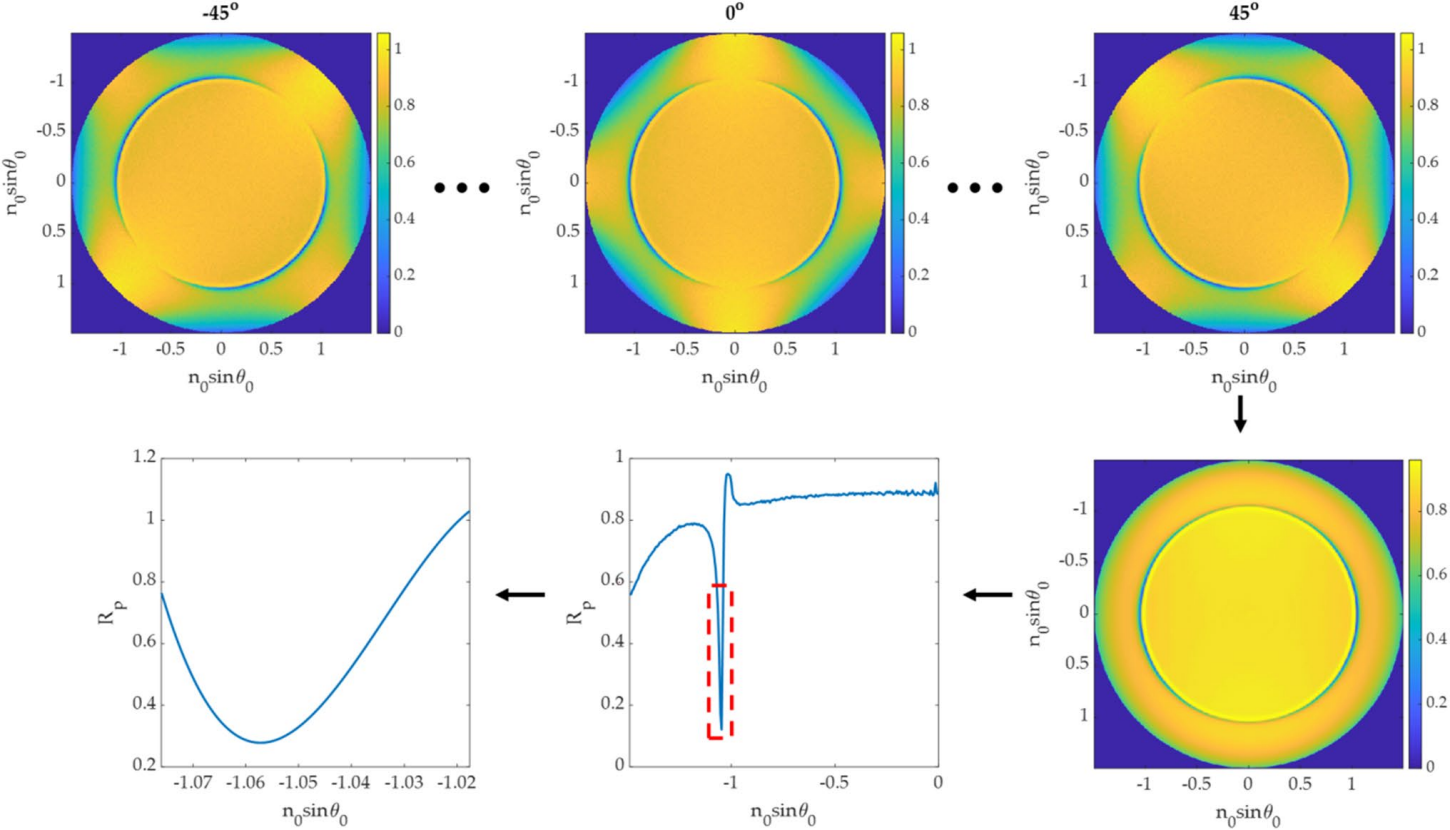
Steps in SPR measurement for azimuthal angle averaging.

Although the pure p-polarization is only in the x-axis in the BFP, the other azimuthal angles (φ) also have a weaker plasmonic effect due to the interference between the p-polarization and the s-polarization. One approach to locate the plasmonic angle and average the noise is to rotate the BFP within the azimuthal angle φ of − 45 degrees to 45 degrees, as shown in Fig. 8. Here the azimuthal angle step size of 1 degree was employed to rotate the BFP image. The 91 line scans along the x-axis for each rotated BFP image were then stored and summed for noise cancellation. The 3rd-degree polynomial curve fitting^10^ was then applied to the summed line-scan to locate the plasmonic angle.

### Quantitative performance parameters

Root mean square phase error of the predicted phase image (${\varnothing }_{RMSE})$ is given as the minimum root mean square error between phase profile predicted from the DL-based method with an arbitrary phase offset (${\varnothing }_{offset}$) and the phase profile computed using Fresnel equation (${\varnothing }_{Fresnel}$) and the transfer matrix approach. The ${\varnothing }_{RMSE}$ is expressed as shown in Eq. (1).

1 $${\varnothing }_{RMSE}=\sqrt{\frac{{\sum }_{j=1}^{{N}_{column}}{\sum }_{i=1}^{{N}_{row}}{({\varnothing }_{DL(i,j)}+{\varnothing }_{offset(i,j)}{-\varnothing }_{Fresnel(i,j)})}^{2}}{{N}_{pixels}}}.$$

where ${\varnothing }_{DL}$ is the phase profile recovered using the DL-based method, ${\varnothing }_{offset}$ is a constant phase shift varying from 0 rad to 2π rad, ${\varnothing }_{Fresnel}$ is the simulated phase profile based on Fresnel equations and the transfer matrix approach, ${N}_{pixels}$ is the total number of pixels; here, the size of the input and the output images are 256 $\times$ 256 pixels, which is 65,536 pixels.

Sensitivity (S) is given by the change in n_0_sinθ_sp_ over the change in sample refractive index (Δn_s_) as expressed in Eq. (2).

2 $$S=\frac{\Delta {n}_{0}\sin{\theta }_{p}}{\Delta {n}_{s}}.$$

Limit of detection (LoD) is given by 3.3 times the standard deviation (3.3σ) of the measurements and expressed in its RIU. The $3.3\sigma$ value corresponds to 99.9% statistical confidence ($\alpha =0.001$). The standard deviation was measured through noise simulation and the Monte Carlo model.

## Results

### Phase responses from the CAN network

**Figure 9:**
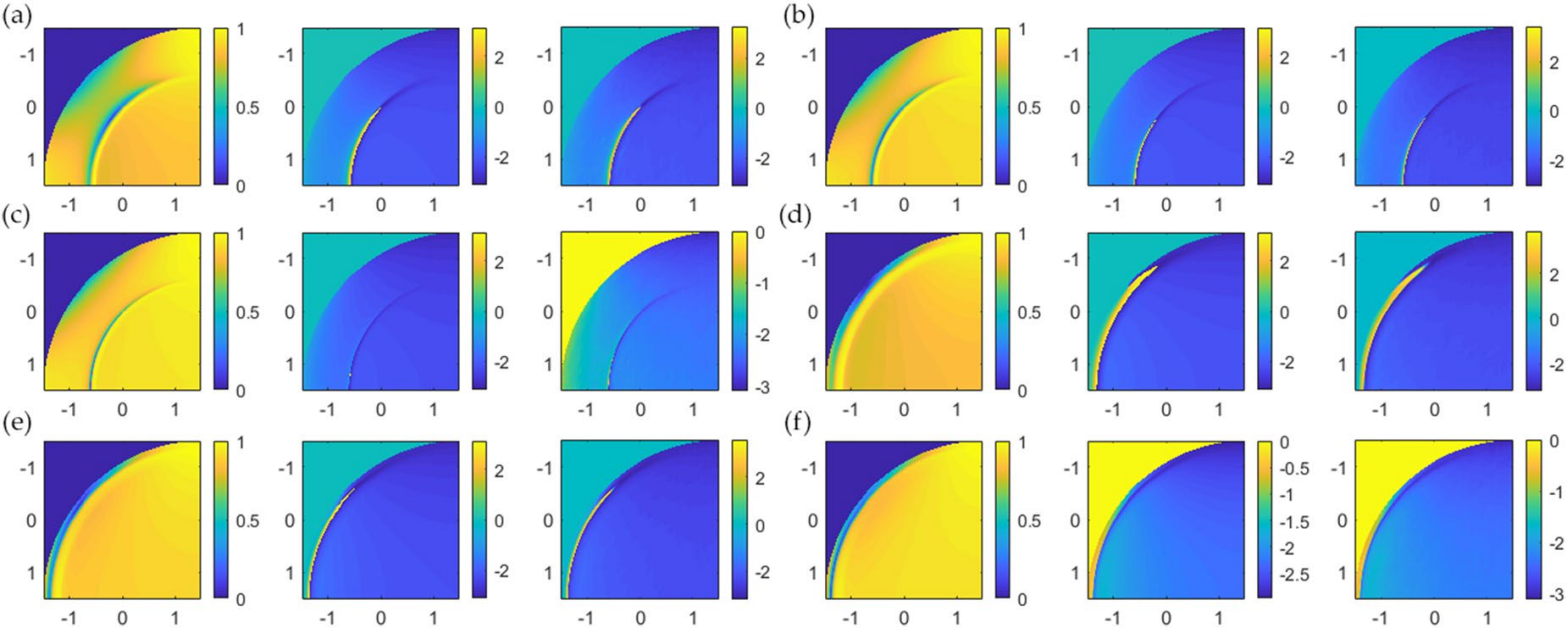
Input/output for the CAN and corresponding phase profiles from Fresnel equations for (**a**) d_m_ of 30 nm and n_s_ of 1.00, (**b**) d_m_ of 40 nm and n_s_ of 1.00, (**c**) d_m_ of 50 nm and n_s_ of 1.00, (**d**) d_m_ of 30 nm and n_s_ of 1.33, (**e**) d_m_ of 40 nm and n_s_ of 1.33, and (**f**) d_m_ of 50 nm and n_s_ of 1.33.

**Figure 10:**
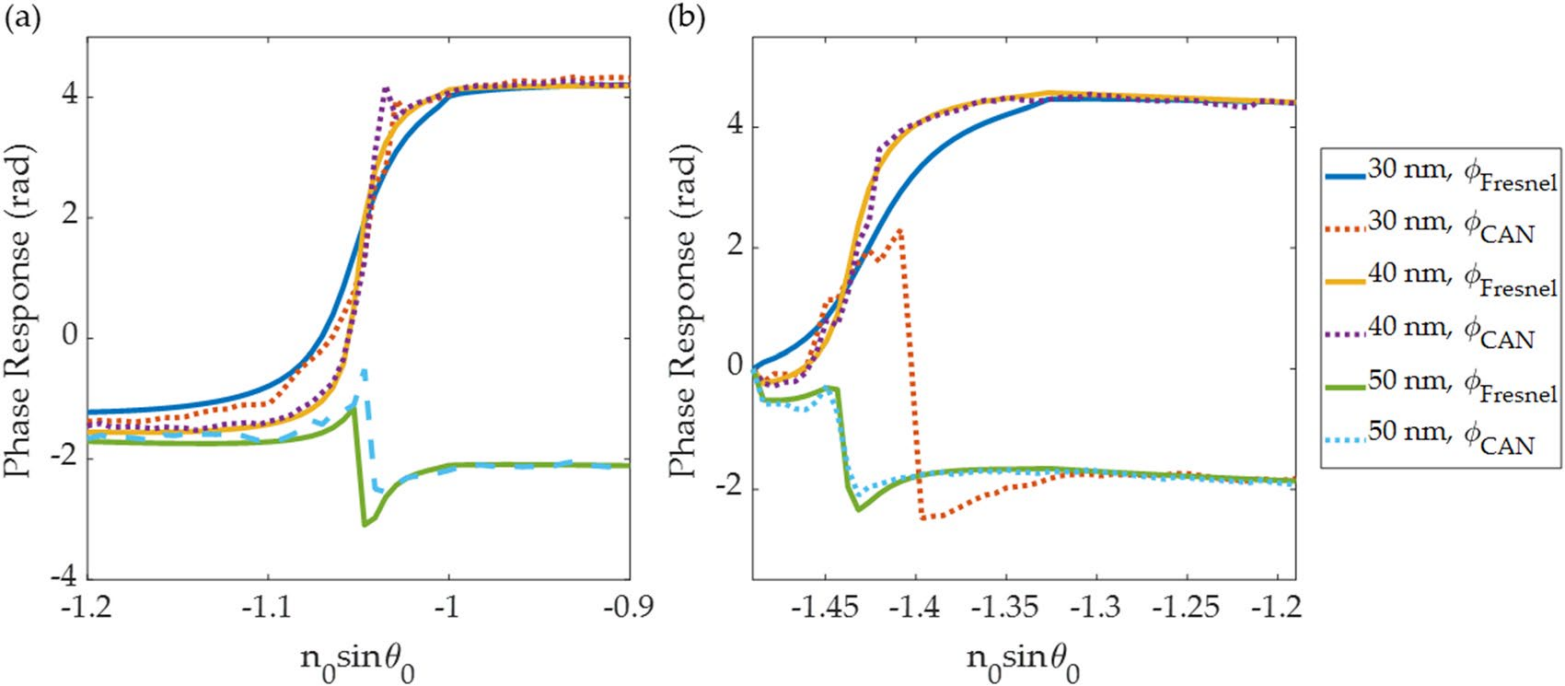
Shows line scans of the simulated phase and the predictive phase profiles in solid curves (the phase from Fresnel calculation) and dash curves (the phase recovered from the CAN). (**a**) n_s_ of 1.00 and (**b**) n_s_ of 1.33.

The CAN network can recover the phase profile for all BFP images in the test dataset, as shown in Fig. 9. The phase can be recovered correctly from a single BFP image, which means the network has recognized the BFP pattern to predict the phase pattern. Figure 10 shows line scans of the phase responses along the x-axis in Fig. 9. The predicted phases agree with the phase profiles calculated using Fresnel equations, although the phases recovered from the CAN have some noise artifacts. The ${\varnothing }_{RMSE}$ errors for single quadrant images were within the range of 0.035 rad (2 degrees) to 0.186 rad (10 degrees) average phase error as shown in Table. 2.

**Table 2:** Root mean square phase error in rad of the test dataset.

Refractive index of the sample	Gold thickness (nm)		
	30	40	50
1.00	0.126	0.078	0.076
1.33	0.189	0.136	0.035

**Figure 11:**
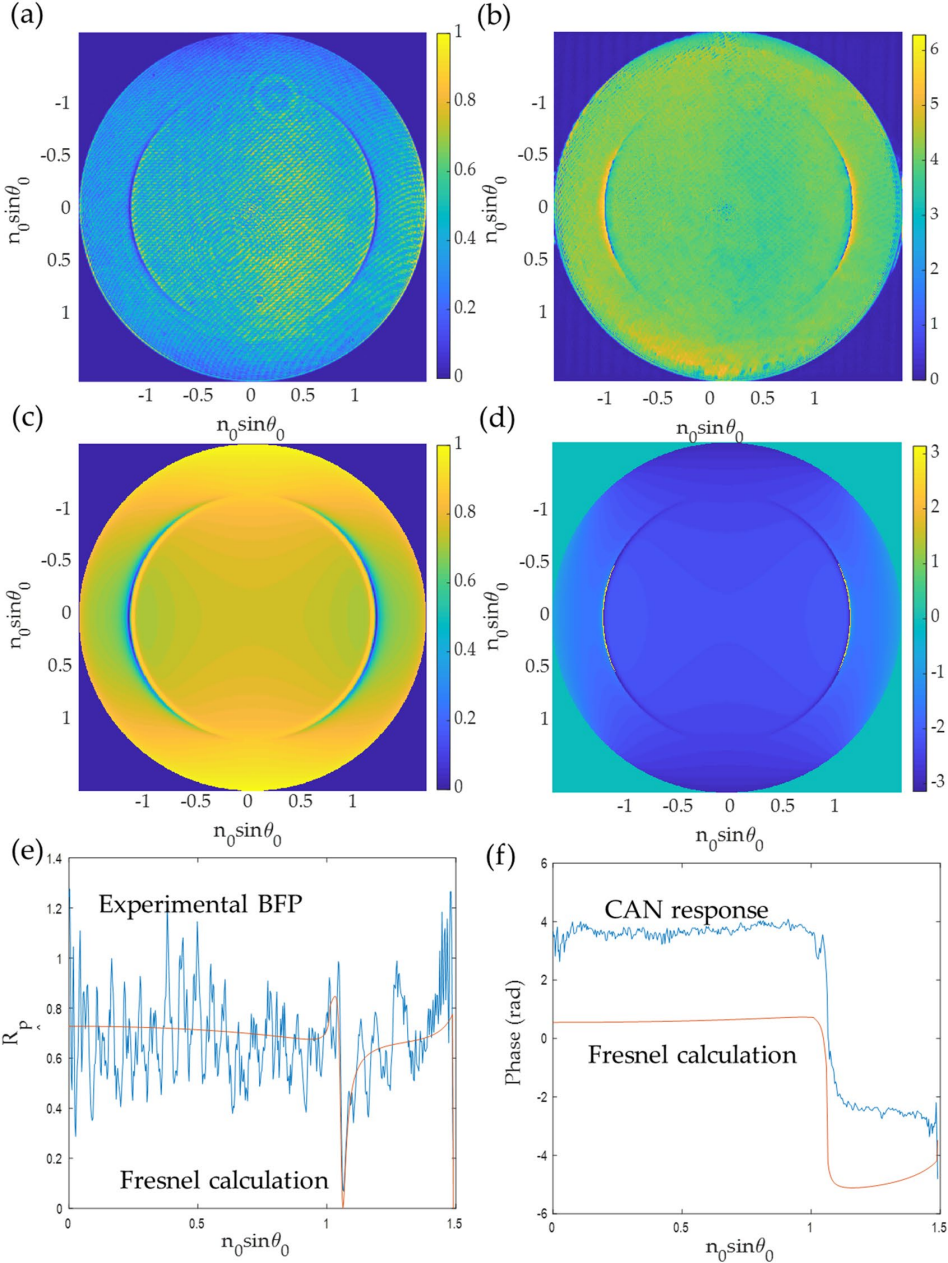
Shows (**a**) experimental BFP for 46 nm of uniform gold layer coated on a BK7 glass substrate (**b**) recovered phase using the CAN. Note that for (**b**), each quadrant of the BFP phase was recovered separately using the CAN, (**c**) simulated BFP intensity using Fresnel equation for 46 nm of uniform gold, (**d**) simulated BFP phase using Fresnel equation, (**e**) BFP intensity line scan from the experimental result and the simulation using Fresnel equation for 46 nm of uniform gold, and (**f**) phase recovered from the experimental result using the CAN and the Fresnel simulation for 46 nm of uniform gold.

Although the CAN network has been trained with simulated data, it can be applied to experimental BFP images. Figure 11a shows an experimental BFP image obtained from the optical microscope setup described in the “Materials and methods” section above and Fig. 2. Figure 11b shows the recovered phase profiles for each quadrant. Figure 11c,d shows simulated BFP intensity and the phase response for 46 nm of a uniform gold layer using Fresnel equations. The plasmonic uniform gold sample tested here was prepared by a sputterer equipped with a quartz microbalance to calibrate the thickness deposition during the sputtering process. The thickness of the gold sensor coated on a standard BK7 microscope coverslip was prepared for 46 nm. Figure 11e,f shows a comparison between the line scans BFP intensity shown in Fig. 11a,c, and phase recovered from the experimental result using the CAN and the Fresnel simulation for 46 nm of uniform gold. It can be seen that the phase recovered from the experimental result agrees with the theoretical phase. Thus, we are of a firm view that simulated data can be generated and utilized as training data; however, a significant concern is how well the simulated data can be generalized to represent the experiment. In this study, the SPR imaging for the gold sensor was generalized by simulation parameter randomization discussed in the “Materials and methods” section.

#### Sensing performance comparison

An essential property of SPR measurement is its capability to provide a quantitative measure of refractive index change in the sensing region. This section demonstrates that the DL-base method can enhance the detection limit for SPR measurement compared to the conventional SPR dip measurement techniques. For SPR refractive index sensing applications, the typical gold thickness is around 50 nm^31^; the 50 nm gold thickness case is investigated in this section.

**Figure 12:**
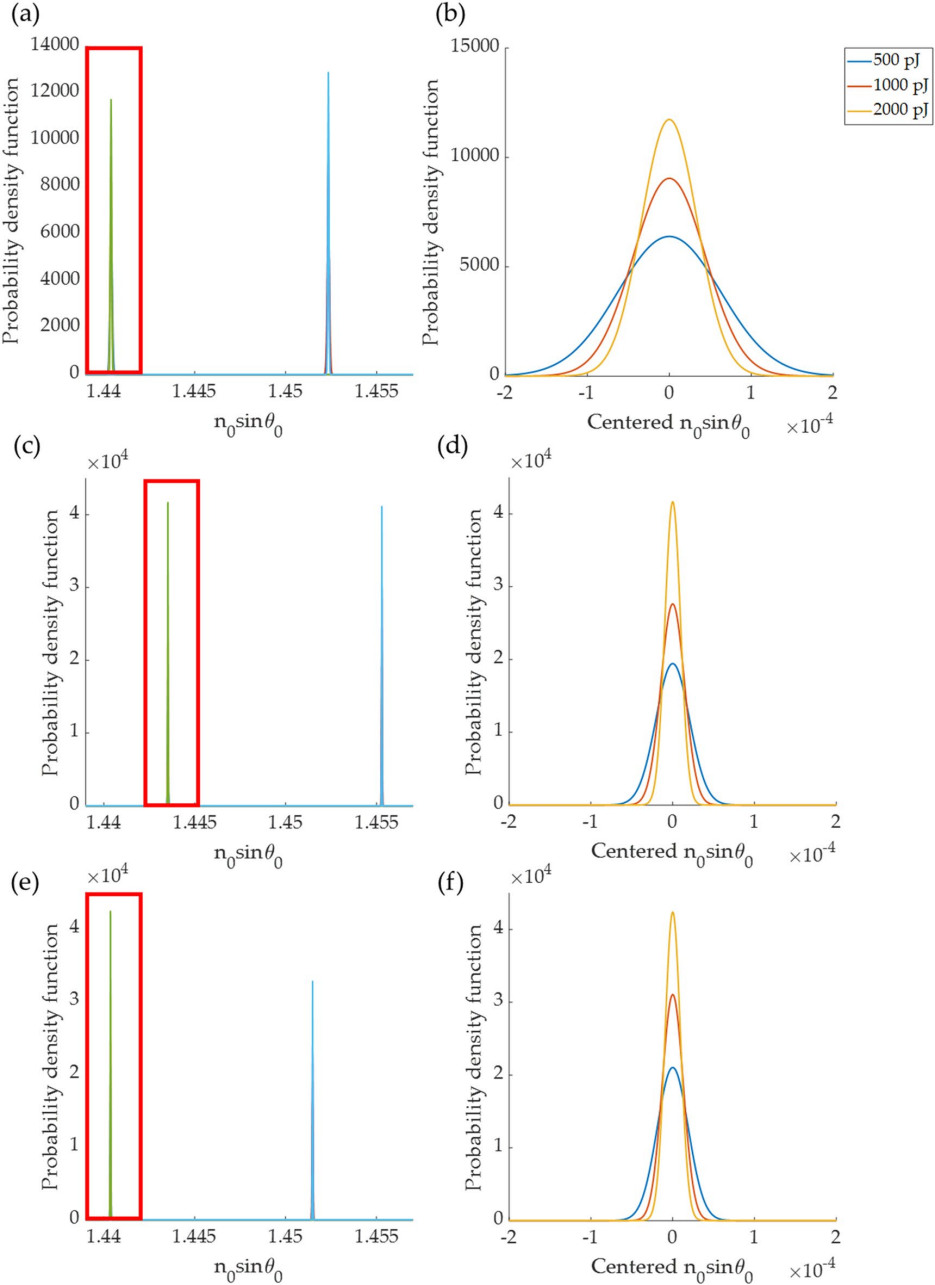
Shows probability density functions of plasmonic dip positions for different photon energy levels of 500 pJ, 1000 pJ, and 2000 pJ calculated for 3 plasmonic dip measurement methods. (**a**) 3rd-degree polynomial curve fitting in intensity line scan image, (**b**) zoomed-in curves of (**a**) in the highlighted curves, (**c**) 45-degree azimuthal averaging, (**d**) zoomed-in curves of (**c**) in the highlighted curves, (**e**) the DL-based method, and (**f**) zoomed-in curves of (**e**) in the highlighted curves.

The total photon energy of the BFP image for the 50 nm gold thickness was varied from 90 to 2600 pJ, and shot noise was added with the corresponding amount of shot noise for the different energy levels. Monte Carlo simulations were carried out to measure the mean and the standard deviation of the three SPR measurement methods described in the “Materials and methods” section. Figure 12. The three measurement methods showed probability density distributions when the sample refractive index was 1.33 and 1.34 at four different total photon energy levels in the BFP image. It can be noticed that the lower light levels gave a broader probability density function leading to an unsatisfactory SPR response. The figure also shows that there is a systematic error in the absolute plasmonic dip position measurement; meanwhile, the change in the plasmonic angle $\Delta {n}_{0}sin{\theta }_{p}$ was the same for all the cases leading to the same sensitivity (*S*) level of 1.1773.

**Figure 13:**
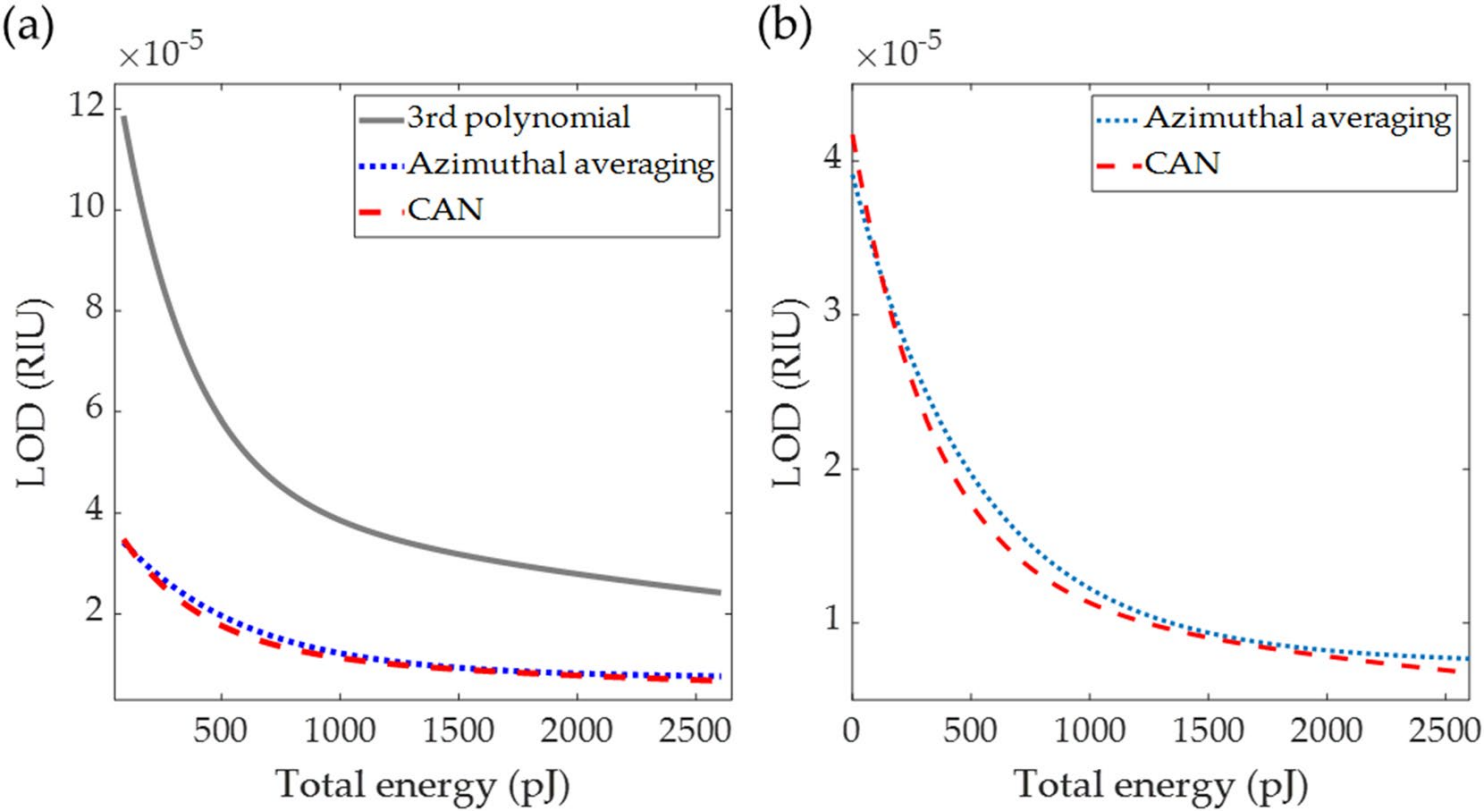
Shows (**a**) detection limits (LOD) in RIU for the three measurement techniques for different incident light energy levels from 90 to 2600 pJ computed for the sample refractive index n_s_ of 1.33, (**b**) zoomed-in figure of (**a**) to compare the CAN method and the azimuthal angle averaging method.

Figure 13 shows the detection limits (LOD) in RIU for the three measurement techniques for different incident light energy levels from 90 to 2600 pJ computed for the sample refractive index n_s_ of 1.33. The LOD for the DL-based method was lower than the 3rd-degree polynomial curve fitting method. The azimuthal angle averaging approach was better than the DL-based method for low light level measurement below 120 pJ. The DL-based performed around 20% better than the azimuthal angle averaging method at a low light energy level of 620 pJ. The DL-based can achieve a similar LOD level to a typical SPR interferometer of 10^–7^ RIU when the total light energy level in the image was 11 nJ, corresponding to the camera well depth of 80,000 electrons. This camera well depth is within the range of the current state-of-the-art sCMOS^50^ and EMCCD^51^ technologies. For the typical camera well depth of 12,000 electrons (equivalent to 1.6 nJ), the DL-based method had 3.5 times lower LOD than the 3rd-degree polynomial curve fitting method and a similar LOD performance azimuthal angle averaging method, respectively. It is crucial to point out that although the LOD performance was similar for a typical well depth camera, the azimuthal angle averaging method does require a reasonable estimation of the image center pixel before applying the image rotation, whereas the other two methods do not. The DL-based method has provided a robust and noise-tolerant method utilizing all the pixels in the BFP for SPR measurement rather than analyzing only some regions around the SPR dip in the image with no need for additional optical components and sophisticated equipment.

## Conclusion

The proposed deep learning-based single-shot phase retrieval method for conventional microscope configuration has been developed. The context aggregation neural network architecture was utilized to predicted phase response for a single-shot microscope image. The network relies on pattern recognition for retrieving the phase profile. We have demonstrated that the simulated data can be employed for training the network. Furthermore, the image dataset can be generalized by parameter randomization. Here, the surface plasmon resonance was employed as an example to quantify the proposed DL-based phase retrieval method. Single quadrant back focal plane images and their corresponding phase images were employed as the input and supervised output for the network. After the training, the network can accurately predict the phase profile of the simulated BFP test dataset and the experimental data with the root mean square phase error below 10 degrees. Here, we have also provided the theoretical framework to analyze the refractive index sensing performance of the proposed DL-based method compared to the SPR dip position measurement methods reported in the literature. For comparison, the 3rd order polynomial curve fitting and the azimuthal angle averaging approaches were simulated to compare the sensitivity and detection limit for different photon energy levels in the image. The sensitivity of the DL-based method is the same as the other intensity detection methods. For low light levels below 120 pJ, the detection limit of the azimuthal angle averaging approach was slightly better than the DL-based method. The detection limit of the azimuthal angle averaging approach outperformed the azimuthal angle averaging for the higher light energy. The detection limit of the DL-based method was 20% better than the azimuthal angle averaging at the light energy level of 620 pJ. For typical cameras with a well depth of 12,000 electrons, the DL-based method performed a 3.5 times better detection limit than the 3rd-degree polynomial curve fitting method. The proposed DL-based method allows us to recover the phase profile of SPR measurement using a conventional microscope configuration through a single-shot BFP image.

## Supplementary Information

Supplementary Information.
