# Supplementary material for: Deep learning-based single-shot phase retrieval algorithm for surface plasmon resonance microscope based refractive index sensing application
Source: Sci Rep. 2021 Aug 11;11:16289. doi: 10.1038/s41598-021-95593-4 (PMC8357982; doi:10.1038/s41598-021-95593-4)
Supplement: Supplementary file 1 — Supplementary Information. [file 41598_2021_95593_MOESM1_ESM.pdf]

Supplementary material

Deep Learning-based Single Shot Phase Retrieval Algorithm for Surface Plasmon Resonance Microscope based Refractive Index Sensing Application

Kitsada Thadson [1], Sarinporn Visitsattapongse [1] and Suejit Pechprasarn [2,3]*

[1] Department of Biomedical Engineering, School of Engineering, King Mongkut's Institute of Technology Ladkrabang, Bangkok 10520, Thailand
[2] Shenzhen Key Laboratory for Micro-Scale Optical Information Technology, Nanophotonics Research Center, Institute of Microscale Optoelectronics, Shenzhen University, Shenzhen 518060, China
[3] College of Biomedical Engineering, Rangsit University, Pathum Thani 12000, Thailand
* Correspondence: suejit.p@rsu.ac.th

Reflection and transmission coefficients of a multilayer structure

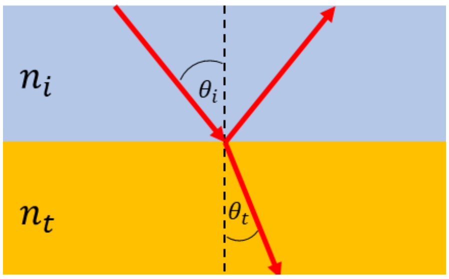

Figure S1. shows the reflectance and transmittance of incident light through different refractive indices of n_i and n_t.

In Fourier optics, Fresnel equation[1] is employed to compute the reflection and transmission coefficients of the different refractive index materials. The linear polarization of incident light is considered in two parts: p-polarization and s-polarization, as shown in eq. (1-2). Fig. S1. shows the reflected light from the interface between the two refractive indices and the propagation through the layer.

$$r_p = \frac{n_i \cos\theta_t - n_t \cos\theta_i}{n_i \cos\theta_t + n_t \cos\theta_i} \quad \text{Eq. 1.}$$

$$r_s = \frac{n_i \cos\theta_t - n_t \cos\theta_i}{n_i \cos\theta_t + n_t \cos\theta_i} \quad \text{Eq. 2.}$$

Where

θ_i is the incident angle in rad.

θ_t is the refractive angle in rad.

n_i is the refractive index of the 1st medium.

n_t is the refractive index of the 2nd medium.

r_p is the reflection coefficient of the p-polarization.

r_s is the reflection coefficient of the s-polarization.

For the multilayer scheme, as shown in Fig. S2., different phases in each layer must be considered for the total reflection and transmission coefficients computation using transfer matrix method[1].

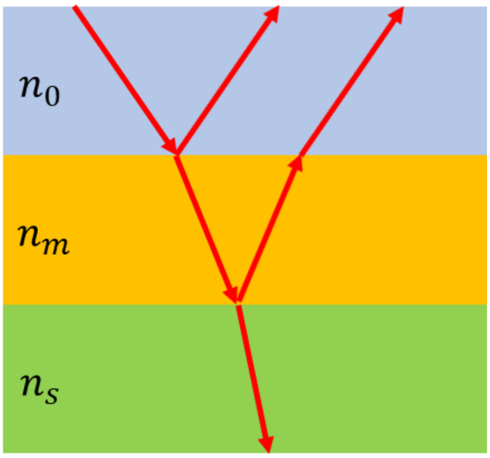

Figure. S2. three layers of the different refractive indices n_0, n_1, and n_2.

The reflection coefficient of three layers can be calculated by:

1. Calculate the reflection coefficient at the interface between n_0 and n_m for the p-polarization (r_{1p}) and the s-polarization (r_{1s}) using eq. (1-2).

2. Calculate the reflection coefficient at the interface between n_m and n_s for the p-polarization (r_{2p}) and the s-polarization (r_{2s}) using eq. (1-2)

3. The phase shift due to the wave propagation inside the n_m layer can be expressed in Eq. 3.

$$\phi_m = \exp\left(\frac{i2\pi n_m d_m \cos\theta_m}{\lambda}\right) \qquad \text{eq. 3.}$$

Note that:

ϕ_m is the phase shift inside the n_m medium in rad.

n_m is the refractive index of the medium.

d_m is the thickness of the n_m medium.

θ_m is the refractive angle through the medium in rad.

λ is the free-space wavelength.

4. The reflection coefficient can be computed using Eq. (4-5) for the p-polarization and the s-polarization, respectively.

$$r_p = \left(\frac{r_{01p}+r_{12p}\phi_m^2}{1+(r_{01p}r_{12p}\phi_m^2)}\right) \qquad \text{Eq. 4.}$$

$$r_s = \left(\frac{r_{01s}+r_{12s}\phi_m^2}{1+(r_{01s}r_{12s}\phi_m^2)}\right) \qquad \text{Eq. 5.}$$

Reference

1 Katsidis, C. C. & Siapkas, D. I. General transfer-matrix method for optical multilayer systems with coherent, partially coherent, and incoherent interference. *Appl. Opt.* **41**, 3978-3987, doi:10.1364/AO.41.003978 (2002).
